# *Ginkgo biloba DFR2* Gene Remodels the Flavonoid Metabolic Network in Transgenic *Nicotiana benthamiana*

**DOI:** 10.3390/plants15091331

**Published:** 2026-04-27

**Authors:** Xinru Sun, Cheng Ji, Pengfei Yu, Guibin Wang, Jing Guo

**Affiliations:** State Key Laboratory of Tree Genetics and Breeding, Co-Innovation Center for Sustainable Forestry in Southern China, Nanjing Forestry University, Nanjing 210037, China; 15663683541@163.com (X.S.); 18479439561@163.com (C.J.); snrqly@163.com (P.Y.); guibinwang99@163.com (G.W.)

**Keywords:** flavonoid metabolism, gene expression, genetic transformation, metabolic flux, regulatory mechanism

## Abstract

Dihydroflavonol 4-reductase (DFR) plays a pivotal role in regulating flavonoid and anthocyanin biosynthesis, governing the accumulation of plant secondary metabolites. This study aimed to characterize the *DFR* gene family in *Ginkgo biloba* and elucidate the function of the predominant gene *GbDFR2* in the flavonoid metabolic network. Through transcriptome analysis, three differentially expressed *GbDFR* genes were identified. Bioinformatic analysis revealed that all three *GbDFR* proteins are hydrophilic and acidic and belong to the NADB_Rossmann superfamily. RT-qPCR analysis of different tissues of ginkgo revealed that all three *GbDFR* genes exhibited the highest expression levels in the leaves. An overexpression vector of *GbDFR2* was constructed and stably transformed into *Nicotiana benthamiana*. Metabolomic and qPCR analyses showed that heterologous *GbDFR2* expression significantly remodeled the flavonoid profile, upregulating sakuranetin and 3,7-Di-*O*-methylquercetin while downregulating narcissin and naringenin chalcone. Additionally, it upregulated endogenous *NbCHI* and *NbDFR*, and suppressed the transcription factors *NbMYL2b* and *NbERF4a*. These findings suggest that *GbDFR2* can act as a regulator of flavonol biosynthesis and provide a candidate gene for the metabolic engineering of flavonoids in woody plants.

## 1. Introduction

Flavonoids represent a large class of plant secondary metabolites with diverse functions in growth, development, and stress responses, among which anthocyanins are responsible for the pigmentation of flowers and fruits [1]. Dihydroflavonol 4-reductase, DFR (EC 1.1.1.219), a canonical NADPH-dependent short-chain dehydrogenase/reductase, governs a critical flux control point in this pathway [2]. It catalyzes the stereospecific reduction of dihydroflavonols (dihydrokaempferol, dihydroquercetin, and dihydromyricetin) to their corresponding leucoanthocyanidins, which are the direct precursors for anthocyanin and proanthocyanidin biosynthesis [2]. Notably, DFR acts downstream of flavanone 3-hydroxylase (F3H), which converts flavanones to dihydroflavonols, underscoring the precise order of the enzymatic steps required for pathway progression [3]. Given its central role, DFR has become a prime target for metabolic engineering to modify pigmentation patterns and increase the accumulation of high-value flavonoid compounds in plants [4,5]. Since the first *DFR* genes were isolated from maize and snapdragon, their orthologs have been extensively characterized across angiosperms and gymnosperms, revealing a high degree of conservation in their core structural domains and catalytic mechanisms despite divergence in nucleotide sequences [6].

*Ginkgo biloba* L., commonly known as the maidenhair tree, represents the sole extant species within the gymnosperm class Ginkgoopsida. Often referred to as a “living fossil”, it occupies a unique evolutionary position and has significant ecological and economic value [7,8]. Historically restricted to glacial refugia in China, *G. biloba* is now widely distributed across temperate and subtropical regions worldwide due to human cultivation [7,9]. *G. biloba* is a cornerstone of traditional Chinese medicine and a major commodity in the global phytopharmaceutical market. Both its leaves and seeds have been utilized in traditional Chinese medicine for millennia [10,11]. The therapeutic efficacy of *G. biloba* leaf extracts is attributed primarily to their unique and abundant constituents: flavonol glycosides and terpene lactones (ginkgolides and bilobalide) [8,10]. Findings also show that *G. biloba* demonstrates remarkable resistance to pests and diseases alongside broad environmental adaptability. *G. biloba* flavonoids demonstrate diverse biological activities, including cardiovascular protection, free radical scavenging, potent antioxidant effects, and potential anticancer properties [12,13,14]. Owing to their distinct structures and bioactivities, these flavonoids and terpene lactones are extensively incorporated into food products and dietary supplements, serving as essential components that underpin the sustainable development of the *G. biloba* industry.

While the flavonoid biosynthetic pathway is well established in model plants, the functional specialization of its regulatory genes, particularly the *DFR* gene family, remains poorly characterized in gymnosperms such as *G. biloba* [8,9,15]. Recent transcriptomic studies have suggested that the expression of *GbDFR* genes is responsive to various environmental and developmental cues, including nitrogen availability, which provides precursors for flavonoid synthesis via phenylalanine [8]. Heterologous expression of certain *GbDFR* genes in tobacco has yielded intriguing but varied phenotypes. For instance, compared with controls, transgenic tobacco heterologously overexpressing the *GbDFR* gene exhibited higher DFR enzyme activity levels and anthocyanin content, along with a darker flower color [1]. However, the ectopic expression of *GbDFR6* induced self-incompatibility-like responses, suggesting functional divergence among family members [16]. The expression of three other *GbDFR* paralogs was tissue-specific (*GbDFR4* in leaves and *GbDFR6* in fruits), and the overexpression of these genes increased DFR activity and anthocyanin levels, while the expression of UV-B and salicylic acid was differentially regulated [17]. In addition, a recent study on the rejuvenation of a 4000-year-old *G. biloba* tree through successive grafting revealed that DFR transcript levels increased but then decreased across grafting generations, coinciding with the changes in total flavonoid content, further supporting the involvement of *GbDFR* genes in growth-phase-dependent metabolic regulation [18]. These findings indicate that the regulatory roles of individual DFR paralogs in shaping the complex flavonoid profile of *G. biloba* are likely distinct and context-dependent [8,19]. A detailed functional analysis of specific *GbDFR* genes is therefore essential to elucidate their precise contributions to flavonol metabolism and to harness their potential for metabolic engineering.

Previous studies have identified several *GbDFR* genes and characterized their expression patterns and heterologous functions in tobacco. The present study builds upon these prior findings by (i) providing a comparative analysis of three GbDFR isoforms, including their tissue-specific expression profiles; (ii) conducting an integrated metabolomic and transcriptomic analysis to identify specific flavonoid metabolites altered by *GbDFR2* overexpression; and (iii) providing a preliminary integration of a multilayer regulatory network involving the flavonoid biosynthesis and metabolism pathway. Our findings not only clarify the regulatory role of *G. biloba DFR2* in flavonoid biosynthesis but also provide a theoretical basis and candidate gene for enhancing the production of secondary metabolites through genetic engineering.

## 2. Materials and Methods

### 2.1. Plant Materials

*Nicotiana benthamiana* plants used for the function study were grown in a controlled-environment chamber at the Laboratory of Nontimber Forest, Nanjing Forestry University. Stably transformed *N. benthamiana* lines were generated from tissue-cultured seedlings maintained in the tissue culture facility at Nanjing Forestry University. Candidate gene identification was performed based on previously generated transcriptome data from *G. biloba* leaves under different nitrogen treatments (SRA accession: SRP574331).

### 2.2. Bioinformatics Analysis

The physicochemical properties of the *GbDFR1*, *GbDFR2*, and *GbDFR3* proteins were analyzed using the ProtParam tool (https://web.expasy.org/protparam/, accessed on 5 October 2024). Hydrophilicity profiles were predicted with ProtScale (https://web.expasy.org/protscale/, accessed on 12 October 2024). Subcellular localization was predicted using CELLO (http://cello.life.nctu.edu.tw/, accessed on 16 October 2024). Conserved motifs were identified using the MEME Suite (https://meme-suite.org/meme/tools/meme, accessed on 20 October 2024) with default parameters. Protein domain architecture was analyzed using the NCBI Conserved Domain Database (https://www.ncbi.nlm.nih.gov/Structure/cdd/wrpsb.cgi, accessed on 2 November 2024) and the SMART online tool (http://smart.embl-heidelberg.de/, accessed on 10 November 2024). Secondary structure predictions were performed using the NPSA server (https://npsa-prabi.ibcp.fr/cgi-bin/npsa_automat.pl?page=/NPSA/npsa_seccons.html, accessed on 5 December 2024), integrating multiple algorithms including MLRC, GOR4, SIMPA96, SOPMA, DSC, and PHD to ensure prediction reliability. Three-dimensional homology models were constructed using SWISS-MODEL (https://swissmodel.expasy.org/, accessed on 20 December 2024).

### 2.3. Analysis of the Tissue-Specific Expression of GbDFR Genes

To investigate the expression profiles of *GbDFR* genes across different tissues, publicly available RNA-seq datasets were retrieved from the European Nucleotide Archive (ENA) database (https://www.ebi.ac.uk/ena/browser/, accessed on 15 February 2025). The following accessions were included: bud, staminate strobilus, and ovulate strobilus (PRJNA289172); root (PRJNA373812); stem (PRJNA473396); cambium (PRJNA488475); developing leaf (PRJNA473396, PRJNA517218, and PRJNA578374), including mutated yellow leaf material (the xantha mutant is called *Ginkgo biloba* ‘Wannianjin’ and obtained a good tree variety certificate (S-SV-GB-008-2014) [20]); and kernel (PRJNA292849). Raw sequencing data were downloaded from the EMBL-EBI database. Quality assessment of the raw reads was performed using FastQC (https://www.bioinformatics.babraham.ac.uk/projects/fastqc/, accessed on 29 February 2025), and adapter sequences and low-quality reads were removed using Trimmomatic [21]. Clean reads were aligned to the *G. biloba* reference genome using STAR [22]. Gene expression levels were quantified as fragments per kilobase of transcript per million mapped reads (FPKM) using RSEM [23]. For visualization, the FPKM values were log2-transformed [log_2_(FPKM + 1)], and a heatmap was generated using the Cloudtutu online platform (http://cloudtutu.com.cn/, accessed on 9 March 2025). Moreover, we extracted RNA from the roots, stems, leaves (green and yellow), ovulate strobilus and staminate strobilus of ginkgo for reverse transcription and performed fluorescence quantitative PCR. The primers used are listed in Table S1.

### 2.4. Vector Construction and N. benthamiana Genetic Transformation

The *GbDFR2* coding sequence was cloned and inserted into the pCAMBIA1302 overexpression vector under the control of the CaMV 35S promoter using homologous recombination and Golden Gate seamless cloning strategies. The recombinant construct was verified by Sanger sequencing. Plasmid DNA was extracted from positive *Escherichia coli* colonies and introduced into *Agrobacterium tumefaciens* strain GV3101 via electroporation. Briefly, 1 μL of purified plasmid (approximately 100 ng) was mixed with 50 μL of GV3101 competent cells, transferred to a prechilled electroporation cuvette, and electroporated under standard conditions. Immediately after electroporation, 1 mL of LB liquid medium was added, and the suspension was transferred to a 1.5 mL microcentrifuge tube. Cells were recovered by incubation at 28 °C with shaking at 180 rpm for 30 min. Subsequently, 50 μL of the culture was spread onto LB agar plates containing appropriate antibiotics and incubated at 28 °C in darkness for 48 h. Positive *Agrobacterium* colonies were confirmed by colony PCR using gene-specific primers, and those producing correctly sized amplicons were selected for tobacco transformation.

For *Agrobacterium*-mediated transformation, sterile *N. benthamiana* leaves were cut into small segments (approximately 0.5 cm × 0.5 cm) and placed on preculture medium for 2–3 days. *Agrobacterium* cultures were pelleted, resuspended in infection medium to an OD600 of 0.2, and used for inoculation. Precultured leaf explants were immersed in the *Agrobacterium* suspension for 10–15 min, blotted dry on sterile filter paper, and transferred to cocultivation medium for 48–72 h in darkness. Following cocultivation, explants were transferred to callus induction medium for approximately 10 days. Well-proliferating calli were subsequently transferred to selection medium containing kanamycin and cultured at 23 ± 2 °C for 15–30 days. Vigorously growing kanamycin-resistant calli were transferred to shoot differentiation medium (4–5 calli per plate) and maintained under a 16 h light/8 h photoperiod at 23 °C for 15–30 days. Regenerated shoots were transferred to rooting medium for 7–10 days. Putative transgenic plants were verified by PCR amplification of the *GbDFR2* fragment from genomic DNA extracted using the CTAB method. Additionally, total RNA was extracted from the leaf tissues of the confirmed positive lines, reverse-transcribed to cDNA, and subjected to quantitative real-time PCR (RT-qPCR) to assess the expression levels of *GbDFR2* across independent transgenic lines. The sequences of the primers used for detection and expression analysis are listed in Table S1.

### 2.5. Flavonoid Extraction and LC-MS/MS Analysis

Flavonoid metabolites were extracted and analyzed using an ultra-performance liquid chromatography–tandem mass spectrometry (UPLC-MS/MS) system consisting of an ExionLC AD UPLC system coupled with a QTRAP 6500+ mass spectrometer (SCIEX, Framingham, MA, USA).

For sample preparation, harvested leaf tissues were immediately frozen in liquid nitrogen and vacuum freeze-dried. Dried samples were ground to a fine powder using a ball mill operated at 30 Hz for 1.5 min. Accurately weighed aliquots (20 mg) of each powdered sample were transferred to centrifuge tubes, and 10 μL of internal standard mixture (4000 nmol/L) was added, followed by the addition of 500 μL of 70% methanol (*v*/*v*) for extraction. The mixtures were ultrasonicated for 30 min and centrifuged at 12,000× *g* for 5 min at 4 °C. The supernatant was collected and filtered through a 0.22 μm membrane filter prior to LC-MS/MS analysis.

Chromatographic separation was performed on a Waters ACQUITY UPLC HSS T3 C18 column (1.8 μm, 100 mm × 2.1 mm i.d.) maintained at 40 °C. The mobile phase consisted of (A) ultrapure water containing 0.05% formic acid and (B) acetonitrile containing 0.05% formic acid, and was delivered at a flow rate of 0.35 mL/min. The injection volume was 2 μL. The elution gradient was programmed as follows: 0 min, 90% A/10% B; 1 min, 80% A/20% B; 9 min, 30% A/70% B; 12.5 min, 5% A/95% B; 13.5 min, 5% A/95% B; 13.6 min, 90% A/10% B; and 15 min, 90% A/10% B.

Mass spectrometric detection was performed using electrospray ionization (ESI) in both positive and negative ion modes. The source temperature was set to 550 °C. The ion spray voltages were 5500 V in positive mode and −4500 V in negative mode. Curtain gas (CUR) was maintained at 35 psi. For each analyte, the declustering potential (DP) and collision energy (CE) were optimized to ensure maximum sensitivity and accuracy. Multiple reaction monitoring (MRM) mode was employed for metabolite quantification.

### 2.6. Differentially Abundant Metabolite Analysis

To identify differentially accumulated metabolites between the transgenic and control plants, orthogonal partial least squares-discriminant analysis (OPLS-DA) was performed. Variable importance in projection (VIP) scores were calculated to assess the contribution of each metabolite to group separation. Metabolites with a VIP > 1 were considered candidate differentially abundant metabolites. Additionally, a fold change (FC) analysis was conducted by comparing the mean metabolite concentrations between groups. Metabolites whose FC was ≥2 (upregulated) or ≤0.5 (downregulated) were retained for further analysis to ensure biological relevance. For functional annotation and pathway enrichment analysis, all the identified metabolites were mapped to the Kyoto Encyclopedia of Genes and Genomes (KEGG) database (http://www.kegg.jp/kegg/compound/, accessed on 15 February 2025). Pathway assignments were retrieved from the KEGG pathway database (http://www.kegg.jp/kegg/pathway.html, accessed on 15 February 2025) to elucidate potential metabolic networks and biological processes affected by *GbDFR2* overexpression.

### 2.7. Statistical Analysis

All the samples used in this experiment were biological replicates, and the three independent lines that displayed the most pronounced overexpression were selected for all subsequent functional studies. One-way analysis of variance (ANOVA) was performed using SPSS 25.0 software (IBM Corp., Armonk, NY, USA) for each group, and Duncan’s multiple comparisons test was used to determine the significance of differences. Origin 2021 software was used to plot and visualize the data.

## 3. Results

### 3.1. Identification and Physicochemical Characterization of Differentially Expressed DFR Genes

Transcriptome analysis of *G. biloba* under different nitrogen treatments revealed five candidate *DFR* genes. Through cross-validation using FPKM quantification and RT-qPCR (|log_2_(fold change)| ≥ 1 and FDR ≤ 0.05), three nitrogen-responsive genes were confirmed and designated *DFR1* (evm.TU.chr12.307), *DFR2* (evm.TU.chr12.306), and *DFR3* (evm.TU.chr12.287). These encode polypeptides of 337 amino acids (*GbDFR1* and *GbDFR2*) and 333 amino acids (*GbDFR3*), with predicted molecular weights of approximately 37 kDa (Table S2). The theoretical isoelectric points ranged from 5.58 (*GbDFR3*) to 6.45 (*GbDFR2*). Charge distribution analysis revealed that *GbDFR3* contained the most acidic residues at 40 Asp + Glu, while *GbDFR2* contained the most basic residues at 34 Arg + Lys. The instability indices ranged from 38.18 to 41.14, with *GbDFR3* exhibiting the highest aliphatic index at 89.55. All the isoforms displayed negative GRAVY values, indicating hydrophilic acidic characteristics. Subcellular localization prediction using the CELLO tool suggested that *GbDFR1* is targeted to the cytoplasm, while *GbDFR2* and *GbDFR3* are predicted to be extracellularly localized. Notably, this prediction differs from the well-established understanding that most plant DFRs are cytoplasmic enzymes involved in flavonoid biosynthesis [24]. Conserved domain analysis classified all three enzymes within the NADB_Rossmann superfamily, establishing them as atypical short-chain dehydrogenase/reductase family members functioning as NADP-dependent reductases essential for flavonoid biosynthesis.

A comprehensive analysis of the secondary structures of the three *GbDFR* proteins revealed that they are primarily composed of alpha helices, extended strands, random coils, and some unidentified regions (Figure S1D). Furthermore, the construction of three-dimensional protein models revealed that for *GbDFR1*, the template with the highest degree of homology was 8fem.1.A, representing the DFR protein from *Panicum virgatum* (switchgrass) in complex with NADP. The model coverage was 94%, with a model similarity of 42%. For both *GbDFR2* and *GbDFR3*, the template with the highest degree of homology was 2nnl.1.A, representing a DFR protein where the binding of two substrate analog molecules alters the functional geometry of the catalytic site. The coverage of the *GbDFR2* model was 94%, and the similarity was 41%, while the coverage of the *GbDFR3* model was 97%, and the similarity was 42% (Figure S1E).

### 3.2. Motif Domain and Phylogenetic Analysis

**Figure 1:**
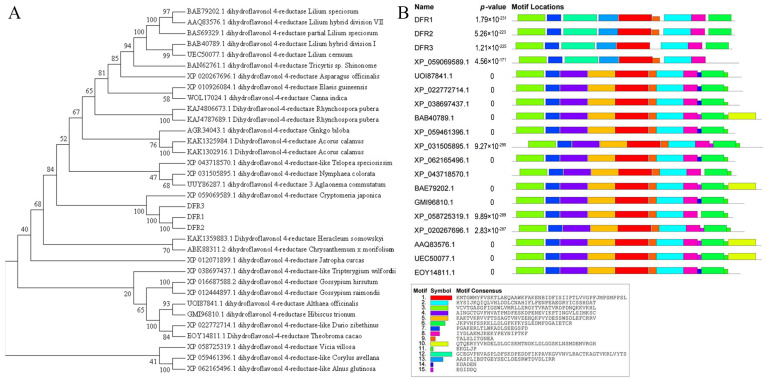
Evolutionary and motif analysis of *G. biloba DFR* proteins. (**A**) Phylogenetic tree construction of *G. biloba* DFRs with homologous sequences from other plant species. (**B**) Conserved motif distribution and sequence analysis of DFR proteins.

Motif prediction analysis revealed that the protein motifs of *GbDFR1* and *GbDFR2* were similar, while that of *GbDFR3* lacked motif 9 (Figure 1B). Phylogenetic analysis showed that the three *GbDFR* proteins clustered within a single clade with dihydroflavonol 4-reductase from *Cryptomeria japonica* (Cupressaceae) (XP_059069589.1), indicating a close evolutionary relationship. These genes were more closely related to dihydroflavonol 4-reductases from *Vicia villosa* (Fabaceae) (XP_058725319.1), *Corylus avellana* (Betulaceae) (XP_059461396.1), and *Alnus glutinosa* (Betulaceae) (XP_062165496.1) (Figure 1A). In summary, *GbDFR1* and *GbDFR2* share the same nine motifs, whereas *GbDFR3* lacks motif 9, which is consistent with the results of the phylogenetic tree.

### 3.3. Tissue-Specific Expression Analysis

**Figure 2:**
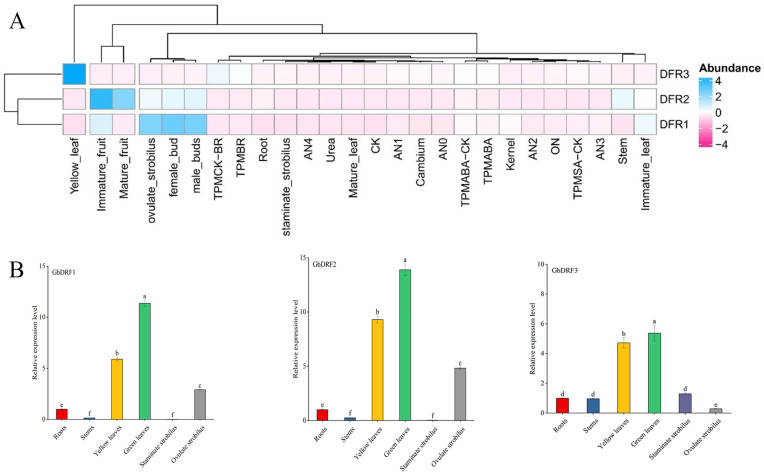
Tissue-specific expression profiles of *GbDFR* paralogous genes in Ginkgo biloba. (**A**) Analysis of the expression patterns of *GbDFRs* in different tissues and under various treatments. Blue indicates increased expression, and pink indicates decreased expression. (**B**) RT-qPCR validation of the relative expression levels of *GbDFR1*, *GbDFR2*, and *GbDFR3* in different ginkgo tissues (root, stem, yellow leaf, green leaf, staminate strobilus, and ovulate strobilus). Different lowercase letters (a, b, c, d, e, f) above the bars indicate statistically significant differences at *p* < 0.05.

Using publicly available *G. biloba* transcriptome datasets, we profiled the spatiotemporal expression dynamics of three *GbDFR* genes across multiple tissues and treatments (Figure 2). The expression of *GbDFR3* was strongly upregulated in response to brassinosteroid treatments (BR1, BR3, and BR4), while the strongest ABA1-induced expression among all the genes was observed during abscisic acid exposure. Notably, *GbDFR1* also significantly responded to ABA. Tissue-specific profiling revealed that *GbDFR3* was maximally expressed in mutated yellow leaves but minimally expressed in immature fruits. Conversely, *GbDFR1* displayed a marked expression predominance in reproductive structures, including female buds, ovulate strobili, and male buds. The expression of *GbDFR2* was detectable in developing fruits, with relatively higher levels in the immature stage than in the mature stage. To further verify the expression levels of the three *DFR* genes in different tissues, we performed RT-qPCR analysis on ginkgo roots, stems, leaves (green and yellow), ovulate strobilus and staminate strobilus. The results indicated that all three *DFR* genes were highly expressed in the leaves.

### 3.4. Genetic Transformation of N. benthamiana and Expression Analysis

**Figure 3:**
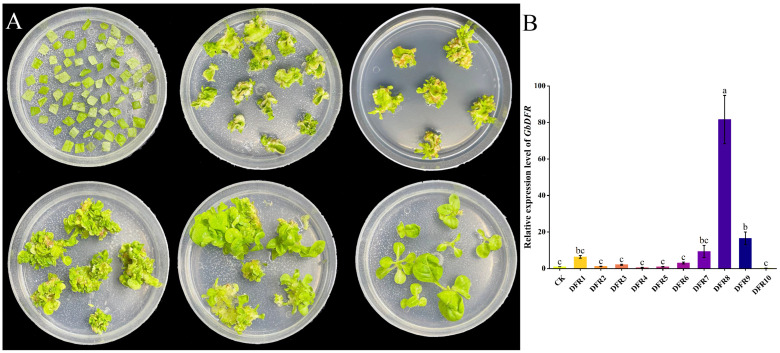
Genetic transformation of *N. benthamiana* with *DFR* and expression analysis in different transgenic lines. (**A**) Schematic diagram of the genetic transformation and screening process using the leaf disc method for *DFR*. (**B**) Analysis of DFR expression levels in different transgenic lines. The vertical bar represents the mean ± SD. The columns with different letters indicate significant differences (*p* < 0.05) in different transgenic lines according to Duncan’s multiple range test.

Due to its responsiveness to different nitrogen forms and expression in leaves being the highest, *GbDFR2* was chosen for further functional exploration. To investigate the biological function of *GbDFR2*, we constructed a stable overexpression vector for the *GbDFR2* gene. This vector was used for genetic transformation of *N. benthamiana* via the leaf disc method. Transgenic plants were obtained following induction, screening, differentiation, and rooting (Figure 3). Expression levels were measured in 10 transgenic *N. benthamiana* lines. Elevated expression of *GbDFR2* was detected in multiple transgenic plants. We selected the three lines with the highest expression levels—designated *DFR1*, *DFR8*, and *DFR9*—as subjects for subsequent studies. These lines underwent targeted quantification of flavonoid metabolites and analysis of the expression levels of key genes in the flavonoid biosynthesis pathway and their associated transcription factors.

### 3.5. Expression Analysis of Flavonoid Biosynthesis Pathway Genes in Transgenic Plants

**Figure 4:**
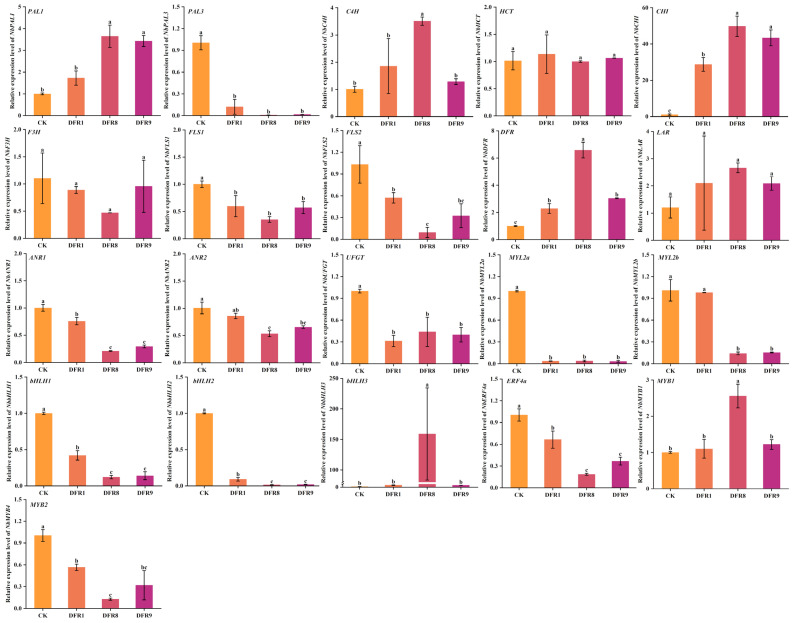
Expression pattern analysis of key genes in three *GbDFR* transgenic lines. Data are presented as the mean ± SD from three independent biological replicates. The columns with different letters indicate significant differences (*p* < 0.05) between different groups according to Duncan’s multiple range test.

To further investigate the specific effects of heterologous *GbDFR2* expression on key enzyme-encoding genes involved in the flavonoid biosynthesis pathway in *N. benthamiana*, we measured the expression of 13 key genes involved in flavonoid synthesis (Figure 4). The results revealed that compared with those in the control group, the expression levels of the *NbCHI* and *NbDFR* genes were significantly upregulated in the transgenic *N. benthamiana* plants. Conversely, the expression of several other genes differed markedly. The expression levels of the key enzyme-encoding genes *NbPAL3*, *NbFLS1*, *NbANR1*, and *NbUFGT* were significantly lower in the transgenic plants than in the wild-type control plants. Furthermore, we examined the expression levels of 8 transcription factors potentially influencing flavonoid synthesis (*NbMYL2a*, *NbMYL2b*, *NbbHLH1*, *NbbHLH2*, *NbbHLH3*, *NbERF4a*, *NbMYB1*, and *NbMYB2*) in transgenic tobacco. Compared with those in the control, the expression levels of *NbMYL2b*, *NbERF4a*, and *NbERF4b* were significantly lower in the transgenic lines.

### 3.6. Flavonoid Metabolite Profiling of GbDFR2-Overexpressing Plants

**Figure 5:**
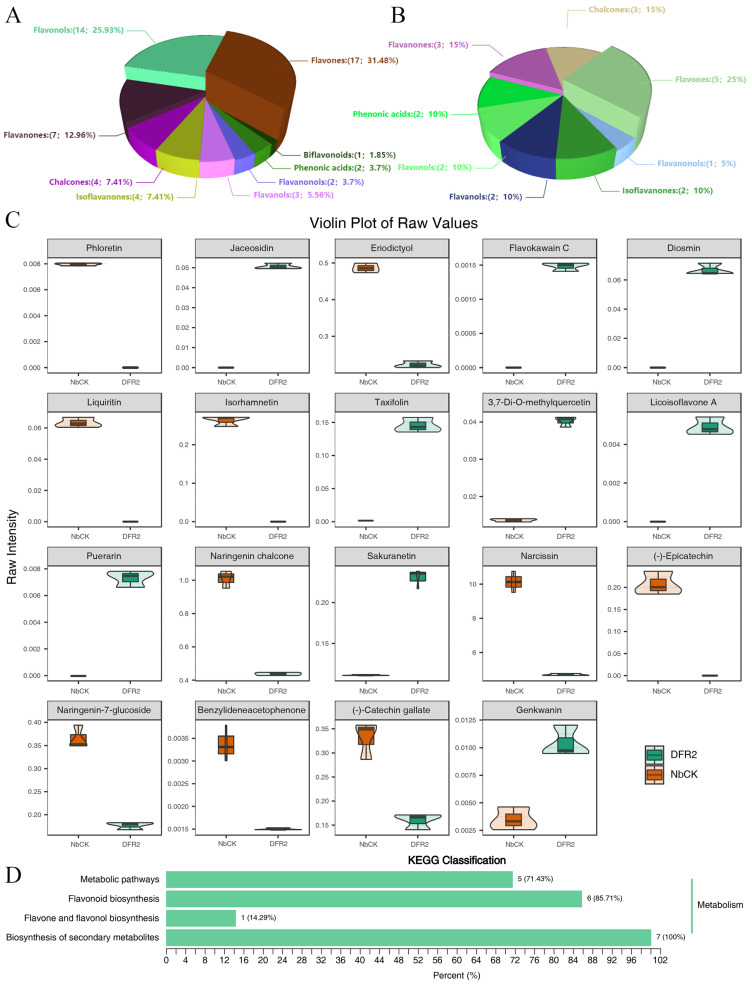
Analysis of flavonoid compounds (**A**) and differentially abundant metabolite categories (**B**), differentially abundant metabolite content (**C**), and enrichment analysis (**D**) in *GbDFR2* transgenic plants. The *DFR2* from the three transgenic lines (*DFR1*, *DFR8*, *DFR9*) was pooled.

Overexpression of *GbDFR2* in *N. benthamiana* enabled the identification of 54 distinct flavonoid compounds through comprehensive metabolomic profiling. These metabolites were systematically classified into ten structural categories: flavanones, flavanonols, biflavonoids, isoflavanones, chalcones, phenolic acids, flavanols, flavones, and flavonols. Quantitative analysis revealed the following distribution: chalcones composed 4 metabolites representing 7.41% of the total flavonoids; flavanones constituted 7 metabolites at 12.96%; flavanonols accounted for 2 metabolites corresponding to 3.70%; phenolic acids included 2 metabolites constituting 3.70%; flavones represented the dominant category, with 17 metabolites at 31.48%; flavonols constituted 14 metabolites, comprising 25.93%; flavanols contained 3 metabolites equivalent to 5.56%; biflavonoids presented 1 metabolite at 1.85%; and isoflavanones consisted of 4 metabolites, representing 7.41% (Figure 5A).

To further compare quantitative metabolite information between different groups, 19 significantly differentially abundant metabolites were identified through comprehensive evaluation using the OPLS-DA model combined with key indicators such as the VIP value and fold change. Among these metabolites, 10 metabolites were downregulated in the transgenic plants, while 9 were upregulated. These differentially abundant metabolites were classified into 8 categories. Chalcones included three differentially abundant metabolites: naringenin chalcone, phloretin, and benzylideneacetophenone, accounting for 15% of the total differentially abundant metabolites. Flavanols contained two metabolites: (-)-catechin gallate and (-)-epicatechin, accounting for 10% of the total. The flavanonol category included taxifolin. The flavanone glycoside category contained naringen-in-7-glucoside, eriodictyol, and liquiritin. Phenolic acids contained flavokawain C. Flavones contained five metabolites: narcissin, sakuranetin, genkwanin, jaceosidin, and diosmin, accounting for 25%. Flavonols contained isorhamnetin and 3,7-Di-*O*-methylquercetin. Isoflavanones contained puerarin and licoisoflavone A, accounting for 10% of the compounds (Figure 5B).

Violin plots of the 19 differentially abundant metabolites revealed distinct expression patterns. Among the upregulated metabolites, the flavonoid sakuranetin exhibited the highest content at 0.23 nmol/g in the DFR group, which was 2.07-fold higher than that in the CK group (Figure 5C). Among the downregulated metabolites, narcissin showed the highest expression at 4.71 nmol/g in the DFR group, representing a decrease of 5.42 nmol/g compared to the CK group (Figure 5C). Additionally, compared with that in the CK group, the flavonoid genkwanin showed the largest upregulation fold-change, followed by that of the flavonol 3,7-Di-*O*-methylquercetin. The chalcone naringenin chalcone showed the most significant downregulation. To elucidate the biological functions and mechanisms of these differentially abundant metabolites, detailed annotation and classification were performed using the KEGG database (Figure 5D). The enrichment analysis indicated that 5 differentially abundant metabolites were associated with metabolic pathways. Six differentially abundant metabolites (naringenin chalcone, eriodictyol, isorhamnetin, sakuranetin, taxifolin, and (-)-epicatechin) were enriched in flavonoid biosynthesis. One differentially abundant metabolite (3,7-Di-*O*-methylquercetin) was enriched in the flavone and flavonol biosynthesis pathway. The biosynthesis of secondary metabolite pathway contained 7 differentially abundant metabolites, representing the highest count and accounting for 100% of the metabolites mapped to this pathway.

### 3.7. Analysis of the Potential Regulatory Mechanism of GbDFR2

**Figure 6:**
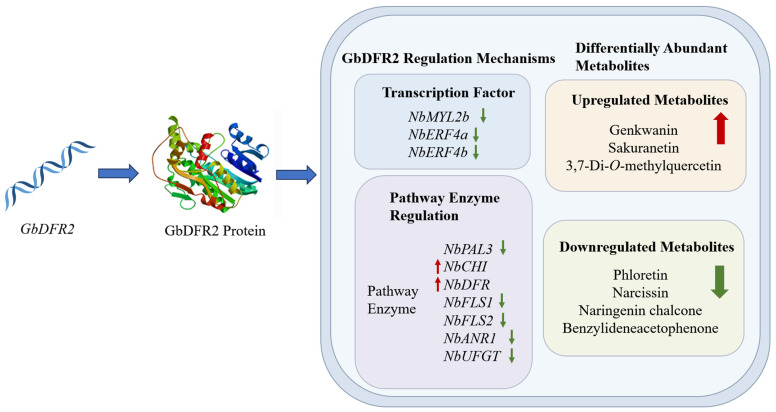
Schematic diagram of the mechanism by which *GbDFR2* regulates flavonoid compound synthesis. Red arrows represent up-regulated expression, and green arrows represent down-regulated expression.

*GbDFR2* regulates the synthesis and accumulation of flavonoids through a multilayered mechanism (Figure 6). First, *GbDFR2* directly catalyzes the conversion of dihydroflavonols to anthocyanin precursors (such as leucocyanidin), directing metabolic flux toward the anthocyanin branch. This led to a significant upregulation of flavone compounds in transgenic tobacco, while chalcone compounds were significantly downregulated due to enhanced upstream enzyme CHI activity. Second, *GbDFR2* influences metabolic flux by regulating transcription factor networks. For instance, the heterologous expression of *GbDFR2* suppressed the expression of transcription factors such as *NbMYL2b* and *NbERF4a*. This may coordinate resource allocation between metabolic branches by inhibiting flavonol branch genes or activating anthocyanin synthesis-related genes. Additionally, *GbDFR2* expression may trigger negative feedback regulation in the phenylpropanoid pathway, as evidenced by the inhibition of *NbPAL3* and *NbFLS1*. The overexpression of the *GbDFR2* gene significantly altered the accumulation patterns of flavonoid metabolites. Metabolomic analysis identified a total of 19 significantly differentially abundant metabolites, with 9 being upregulated and 10 being downregulated. The most pronounced upregulated metabolites were the flavone sakuranetin and the flavonol 3,7-Di-*O*-methylquercetin, while the flavone genkwanin exhibited the highest fold-change increase. The most significantly downregulated metabolites included the flavone narcissin and the chalcone naringenin chalcone. In summary, *GbDFR2* dynamically regulates the synthesis and accumulation of flavonoids, providing new insights for deciphering the complex regulatory mechanisms of plant secondary metabolism.

## 4. Discussion

The *DFR* genes in *G. biloba* exhibit marked functional divergence and tissue-specific expression patterns, reflecting their specialized roles in flavonoid metabolism. The high expression of *GbDFR3* in mutated yellow leaves (Figure 2A) suggests its potential involvement in antioxidant defense mechanisms during the stress response, which is consistent with the established role of flavonoids in reactive oxygen species scavenging [1]. Furthermore, the high expression of *GbDFR3* in yellow leaf mutants may also contribute to leaf color variation and enhance photoprotection under high-light or stress conditions by promoting the accumulation of flavonoids and anthocyanins, thereby alleviating photoinhibition [20]. Notably, the predicted extracellular localization of *GbDFR2* and *GbDFR3* differed from the typical cytoplasmic localization of most plant DFRs [24]. However, as this prediction is based solely on in silico analysis, experimental validation (e.g., via GFP fusion expression) is necessary before any functional interpretation can be proposed. The expression levels of three *GbDFR* genes were high in the leaves (Figure 2B), indicating that this gene may have a major regulatory effect on the leaves of ginkgo.

Bioinformatic analysis confirmed that all three *GbDFR* proteins belong to the NADB_Rossmann superfamily (EC 1.1.1.219) and are classified as atypical members of the short chain dehydrogenase/reductase (SDR) family. Their conserved domains are intimately associated with NADP-dependent flavonoid reduction. Motif analysis revealed that *GbDFR1* and *GbDFR2* share nine conserved motifs, in which *GbDFR3* lacks motif 9, a structural divergence reflected in the phylogenetic tree where *GbDFR3* clusters separately from *Cryptomeria japonica* DFR. These findings align with those of studies on strawberry DFR, which demonstrated that the Rossmann fold domain is critical for NADPH binding [2]. The absence of specific motifs may perturb the tertiary structure and substrate binding capacity, potentially contributing to functional divergence among family members. Evolutionary analysis further indicated significant divergence between *GbDFRs* and those from Fabaceae and Betulaceae species, reflecting the evolutionary specialization of flavonoid metabolic regulation between gymnosperms and angiosperms [1,4].

Compared with the control, the transgenic lines accumulated substantially higher levels of the flavone sakuranetin and the flavonol 3,7-Di-*O*-methylquercetin, which is consistent with the *DFR2*-mediated redirection of metabolic flux toward anthocyanin precursors, consequently limiting substrate availability for competing flavonol biosynthesis branches. This observation aligns with reports that *DFR* overexpression induces anthocyanin accumulation in grape (*Vitis vinifera*) [5]. Notably, the downregulation of naringenin chalcone suggests concomitant activation of upstream chalcone isomerase (CHI) activity, revealing bidirectional metabolic regulation. Furthermore, the suppression of the phenylpropanoid pathway genes *NbPAL3* and *NbFLS1* may represent a negative feedback mechanism balancing flavonoid and other secondary metabolite fluxes—a widespread strategy in plant metabolic resource allocation [3]. In addition, the complexity of the flavonoid biosynthesis pathway is reflected in its multilayered regulation by transcription factors. In this study, heterologous expression of *GbDFR2* led to significant downregulation of the expression of transcription factors such as *NbMYL2b* and *NbERF4a* in tobacco. This likely suppresses the formation of MYB-bHLH-WD40 (MBW) transcriptional complexes, thereby reducing the expression of flavonol branch genes. In parallel, the observed upregulation of sakuranetin in transgenic lines correlates with increased expression of NbCHI, an enzyme that accelerates the conversion of naringenin chalcone to naringenin, the direct precursor of sakuranetin [25,26]. This is supported by the concurrent downregulation of naringenin chalcone and elevated sakuranetin accumulation. Similarly, the downregulation of *NbANR1* suggests a metabolic flux shift toward anthocyanin precursors and away from the proanthocyanidin branch. Such interspecies regulatory differences may stem from incompatibility between *GbDFR* substrate selectivity and the endogenous regulatory network of tobacco, indicating significant species-specific functional evolution of DFR orthologs [13].

Overexpression of *DFR* genes universally modulates flavonoid pathways across plant species, yet elicits divergent metabolic outcomes contingent upon species-specific factors and gene characteristics. While *DFR* typically promotes anthocyanin and proanthocyanidin accumulation [27,28], this study revealed that *GbDFR2* overexpression preferentially upregulated sakuranetin and 3,7-Di-*O*-methylquercetin while significantly downregulating narcissin and naringenin chalcone. Such isoform-specific effects align with engineering successes in other systems: active site modification of *Petunia hybrida* DFR enabled pelargonidin-type anthocyanin biosynthesis [29], while heterologous expression of *Camellia sinensis CsDFRa/CsDFRc* restored anthocyanin synthesis in Arabidopsis mutants [27]. Notable exceptions exist; however, RNA silencing of chrysanthemum *DFR* paradoxically increased total flavonoids through feedback upregulation [30], whereas *GbDFR6* overexpression in tobacco induced anthocyanin accumulation alongside developmental defects and self-incompatibility phenotypes, potentially through hormonal interference [19].

The results of this study suggest that *GbDFR2* can modulate flavonoid biosynthesis through integrated transcriptional and metabolic reprogramming. These findings deepen our understanding of plant secondary metabolic regulation and provide a theoretical foundation for the use of genetic engineering to increase the medicinal value of *G. biloba* or develop novel cultivars with modified flavonoid profiles. However, genetic transformation of woody plants such as *G. biloba* remains challenging due to recalcitrant regeneration and low transformation efficiency. In the present study, heterologous expression in *N. benthamiana* served as an alternative strategy to circumvent these limitations and investigate the regulatory function of *GbDFR2*. Therefore, the observed metabolic remodeling should be interpreted as the functional potential of *GbDFR2* in a heterologous system, whereas its native biological role in *G. biloba* awaits further validation through homologous transformation. To directly validate the role of *GbDFR2* in ginkgo, future efforts should focus on establishing an efficient stable transformation system for ginkgo, employing *Agrobacterium*-mediated transformation of embryonic calli, or developing overexpression and CRISPR/Cas9-based gene editing techniques in ginkgo suspended cells. Furthermore, further investigations are also needed to validate the predicted extracellular localization of specific *GbDFR* isoforms, elucidate the structural basis of their substrate specificity through protein crystallography or mutagenesis approaches, and explore the potential of *GbDFR2* as a biotechnological tool for metabolic engineering in medicinal trees.

## 5. Conclusions

This study characterized three *GbDFR* isoforms from *G. biloba* and demonstrated that heterologous expression of the *GbDFR2* remodeled the flavonoid metabolome in *N. benthamiana*, upregulating sakuranetin and 3,7-Di-*O*-methylquercetin while downregulating narcissin and naringenin chalcone. These changes correlate with altered expression of endogenous *NbCHI* and *NbDFR* and transcription factors *NbMYL2b* and *NbERF4a*. Our findings suggest that *GbDFR2* can modulate flavonoid profiles in a heterologous system and may serve as a candidate gene for future metabolic engineering efforts in woody plants.

## Data Availability

The data supporting the conclusions of this study are included within the article.

## Supplementary Materials

The following supporting information can be downloaded at: https://www.mdpi.com/article/10.3390/plants15091331/s1, Figure S1. The structural analysis of *G. biloba* DFR proteins. (A) Transmembrane domain prediction of DFR proteins; (B) Conserved domain of the DFR proteins; (C) Pfam NmrA domain (PF05368); (D) Secondary structure analysis of DFR proteins; (E) Tertiary structure prediction of DFR proteins. Table S1. Primer Sequence Information. Table S2. Physicochemical properties and subcellular localization of DFR isoforms.
