# Supplementary material for: Ginkgo biloba DFR2 Gene Remodels the Flavonoid Metabolic Network in Transgenic Nicotiana benthamiana
Source: Plants (Basel). 2026 Apr 27;15(9):1331. doi: 10.3390/plants15091331 (PMC13164805; doi:10.3390/plants15091331)
Supplement: Supplementary file 1 [file plants-15-01331-s001.zip › plants-4226728-supplementary.pdf]

Table S1. Primer Sequence Information.

Name	Forward primer	Reverse primer
GbDFR2-ORF	ATGGGCGACCGACAACGTAAG	CTATTGCAACACTCCCATCATGATGGC
GbDFR2-qPCR	GGCCCTAGTGGGTGGACCTT	GCCCAACACATGTCGAAGCG
GbDFR1-qPCR	GGGTGGACCTTGCCTTACCC	GCCCAACACATGTCGAAGCG
GbDFR3-qPCR	TCGCTTTCCGCAGCTTCTCC	TACATCCACGAGGGCGGCTT
GbGAPDH	GGTGCCAAAAAGGTGGTCAT	CAACAACGAACATGGGAGCAT
18T-seq	GATGTGCTGCAAGGCGATTAAG	CTTTATGCTTCCGGCTCGTATG
NbActin	CTTGAAACAGCAAAGACCAGC	CATCCTATCAGCAATGCCCG
NbPAL1	TGGGAAGCCAGCAATTCCTA	GGTGATTGGACTTTCTCGCC
NbPAL3	CGCGCTGCAACTAAGATGAT	GATAGGGGTGCCTTGGAAGT
NbC4H	TTTTGTTGACGAGAGGAAGAAGC	GTCCTCGTTGATTTCTCCCTTTT
NbHCT	ATGCATACTCGAGTTGTTGAGCA	CCGAGCAACTTGGTAAGATTCAC
NbCHI	CAATGGTGCATTCATATCCTTCA	AATTGACCGGTGACAACATCTCT
NbF3H	CTACTCAAGGTGGCCAAACAATC	ACTTTCTGGTCCATGTCAACACA
NbFLS1	ATGCATACTCGAGTTGTTGAGCA	CCGAGCAACTTGGTAAGATTCAC
NbFLS2	GAACTTGAAGGGAAAAGGGG	TCCCTGTAGGAGGGAGGATT
NbDFR	AACCAACAGTCAGGGGAATG	TTGGACATCGACAGTTCCAG
NbLAR	AGGTCCTTTACGCCATC	ACGAACCTGCTTCTCTTTGG
NbANR1	CATTTGACTTTCCCAAACGC	ATTGGGCTTTTGAGTTGTGC
NbANR2	TTCCCACTTGGGATGATA	CACCTATACTCTGTTAGTGGC
NbUFGT	GAGTGCATTGGATGCCTTTT	CCAGCTCCATTAGGTCCTTG
NbMYL2a	GTCTCATTTTGGTGCAGGGG	TCTGGAAGCAGGTGACCTTT
NbMYL2b	GGGGCACAACAAGTTCAGTT	ACGCCATTGTTCTGAGCTTC
NbbHLH1	CTATCCACAGCAAACCAACAC	TCAAATACTCTCATCAGCAGAC
NbbHLH2	CAGGGAGAAATTGAGGAGAG	GGGTAACTTATAATCGGCAC
NbbHLH3	TTACTGATGAAAGCCGTGAG	TGATTGCTGAAGAAAGGATAGG
NbERF4a	TAGCGGAGAGACTGGCATTC	GTCGGCTGTTGGTGGAAAAT
NbMYB1	ATTAGTCCTCCTCATGACCA	TAGACCATTAGCCTTCAACC
NbMYB2	TTGAGTCGTGGTATTGATCCT	CTGAGCTCAAGATTTAAGTCAGG

Table S2. Physicochemical properties and subcellular localization of *DFR* isoforms.

Name	ID	ORF Length	Protein Length	Molecular Mass (kDa)	pI	Total Negatively Charged Residues （Asp+Glu）	Total Positively Charged Residues (Arg + Lys)	Instability Index	Aliphatic Index	GRAVY	Subcellular Localization
DFR1	evm.TU.chr12.307	1014	337	36.8703	5.66	39	32	38.18	86.8	-0.092	Cytoplasm
DFR2	evm.TU.chr12.306	1014	337	36.65416	6.45	36	34	38.88	88.52	-0.099	Extracellular
DFR3	evm.TU.chr12.287	1002	333	36.79914	5.58	40	31	41.13	89.55	-0.133	Extracellular

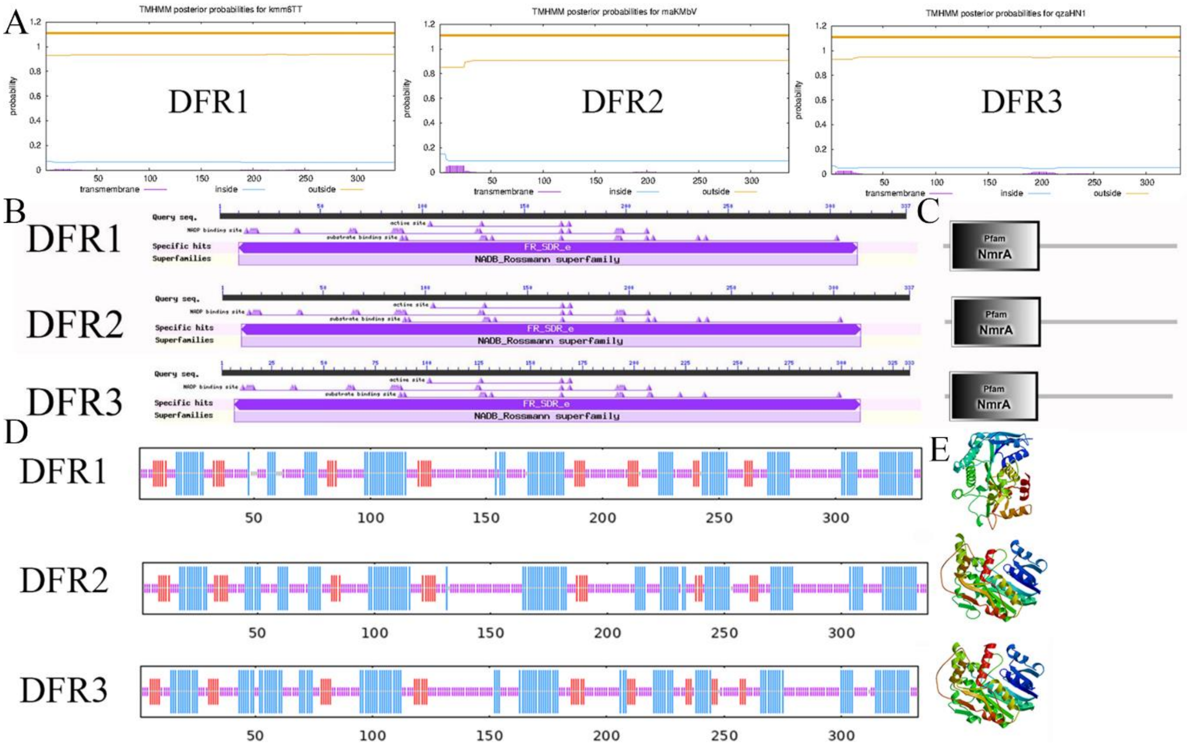

Figure S1. The structural analysis of *G. biloba* DFR proteins. A. Transmembrane domain prediction of DFR proteins; B. Conserved domain of the DFR proteins; C. Pfam NmrA domain (PF05368); D. Secondary structure analysis of DFR proteins; E. Tertiary structure prediction of DFR proteins.
